# Circular RNA ACVR2A suppresses bladder cancer cells proliferation and metastasis through miR-626/EYA4 axis

**DOI:** 10.1186/s12943-019-1025-z

**Published:** 2019-05-17

**Authors:** Wei Dong, Junming Bi, Hongwei Liu, Dong Yan, Qingqing He, Qianghua Zhou, Qiong Wang, Ruihui Xie, Yinjie Su, Meihua Yang, Tianxin Lin, Jian Huang

**Affiliations:** 1 Department of Urology, Sun Yat-sen Memorial Hospital, Sun Yat-sen University, 107th Yanjiangxi Road, Guangzhou, China; 2 Guangdong Provincial Key Laboratory of Malignant Tumor Epigenetics and Gene Regulation, Sun Yat-sen Memorial Hospital, Sun Yat-sen University, 107th Yanjiangxi Road, Guangzhou, China

**Keywords:** circACVR2A, miRNA-626, EYA4, Proliferation, Metastasis, Bladder cancer

## Abstract

**Background:**

Circular RNAs (circRNAs) have been considered to mediate occurrence and development of human cancers, generally acting as microRNA (miRNA) sponges to regulate downstream genes expression. However, the aberrant expression profile and dysfunction of circRNAs in human bladder cancer remain to be investigated. The present study aims to elucidate the potential role and molecular mechanism of circACVR2A in regulating the proliferation and metastasis of bladder cancer.

**Methods:**

circACVR2A (hsa_circ_0001073) was identified by RNA-sequencing and validated by quantitative real-time polymerase chain reaction and agarose gel electrophoresis. The role of circACVR2A in bladder cancer was assessed both in vitro and in vivo. Biotin-coupled probe pull down assay, biotin-coupled microRNA capture, dual-luciferase reporter assay, and fluorescence in situ hybridization were conducted to evaluate the interaction between circACVR2A and microRNAs.

**Results:**

The expression of circACVR2A was lower in bladder cancer tissues and cell lines. The down-regulation of circACVR2A was positively correlated with aggressive clinicopathological characteristics, and circACVR2A served as an independent risk factor for overall survival in bladder cancer patients after cystectomy. Our in vivo and in vitro data indicated that circACVR2A suppressed the proliferation, migration and invasion of bladder cancer cells. Mechanistically, we found that circACVR2A could directly interact with miR-626 and act as a miRNA sponge to regulate EYA4 expression.

**Conclusions:**

circACVR2A functions as a tumor suppressor to inhibit bladder cancer cell proliferation and metastasis through miR-626/EYA4 axis, suggesting that circACVR2A is a potential prognostic biomarker and therapeutic target for bladder cancer.

**Electronic supplementary material:**

The online version of this article (10.1186/s12943-019-1025-z) contains supplementary material, which is available to authorized users.

## Background

Bladder cancer (BC) ranks as the ninth most frequently-diagnosed cancer worldwide, and it’s the most common malignancy of urinary tract with high morbidity and mortality rates [1]. BC can be divided into two groups according to its distinct behavior: low-grade non-muscle-invasive bladder cancer (NMIBC) and high-grade muscle-invasive bladder cancer (MIBC). Although NMIBC is usually treatable by transurethral resection and intravesical therapy, it’s more likely to relapse and progress to MIBC [2]. MIBC frequently develops lymph node (LN) metastasis and distant metastasis and leads to poor prognosis [3]. Metastasis is life-threatening, and the 5-year survival rate is only 8.1% [4]. Nevertheless, there are no effective therapeutic methods for BC patients with tumor relapse or metastasis. Therefore, the molecular mechanisms that promote BC development and progression are essential for further study.

Circular RNAs (circRNAs) represent a novel class of non-coding RNAs characterized by a covalently closed loop without 5′ cap and 3′ polyadenylated tail [5], and they are derived from exon ‘skipping’ and ‘direct back-splicing’ of pre-mRNA transcripts [6]. Currently, with the development of bioinformatics analysis and high throughput sequencing, a plenty of circRNAs have been identified in mammalian cells [7–9]. Emerging evidence indicates that circRNAs are involved in the regulation of gene transcription and translation, and in the cytoplasm and nuclear localization of proteins, suggesting that they may participate in the progression of many diseases, including cancers. CircRNAs can function as microRNA (miRNA) sponges, RNA-binding protein sponges and protein-coding genes. For instance, ciRS-7 promotes colorectal cancer progression by blocking of tumor suppressive effects of miR-7 [10]. CircEPSTI1 affects triple-negative breast cancer proliferation and apoptosis through sponging miR-4753 and miR-6809 [11]. Circ-Foxo3 arrests CDK2 and blocks cell cycle progression by forming the circ-Foxo3-p21-CDK2 ternary complex [12]. Circ-Amotl1 reduces apoptosis and enhances cardiac repair by binding to PDK1 and AKT1, activating AKT phosphorylation and nuclear translocation [13]. Circ-ZNF609 is associated with heavy polysomes, and can be translated into a protein in a splicing-dependent and cap-independent manner [14]. An 87-amino-acid peptide encoded by the circular form of LINC-PINT directly interacts with polymerase associated factor complex (PAF1c) and suppresses the transcriptional elongation of multiple oncogenes of glioblastoma [15]. Recently, several circRNAs have been reported to be aberrantly expressed in BC cell lines and tissues, and they regulate proliferation, apoptosis, metastasis and epithelial-mesenchymal transition (EMT) [16–19]. However, the biological functions and clinical significance of circRNAs implicated in BC remain largely unknown.

In our present research, we identified a novel tumor suppressor circACVR2A from a published RNA sequencing (RNA-seq) data of human BC tissues and pair-matched normal bladder tissues, and verified the results using our established BC cell lines metastasis model [18, 20, 21]. We revealed that circACVR2A was derived from exons 3, 4 and 5 of the ACVR2A gene, and was significantly down-regulated in BC tissues and cell lines. BC patients with lower expression of circACVR2A were positively associated with advanced pathological stage, high grade, lymphatic metastasis and poorer survival. Furthermore, we demonstrated that circACVR2A could inhibit proliferation, migration and invasion of BC cells via binding to miR-626 as a miRNA sponge to regulate EYA4 expression. Therefore, circACVR2A may be a promising independent prognostic biomarker and potential target in BC therapy.

## Methods

### Patient tissue specimens

BC tissues and matched adjacent normal epithelial tissues were obtained from patients who underwent surgery at the Department of Urology of Sun Yat-sen Memorial Hospital (Guangzhou, China). All tissue specimens were immediately frozen in liquid nitrogen after surgical removal and stored at − 80 °C until RNA extraction. Histological and pathological diagnoses were independently confirmed by two experienced pathologists. The use of human BC tissue specimens was evaluated and approved by the Ethical Committee of Sun Yat-sen Memorial Hospital, and written informed consent was obtained from all patients.

### Cell culture and treatments

Human BC cell lines T24, UM-UC-3, RT4, J82, 5637, HT-1376, TCCSUP and the immortalized normal uroepithelium cell line SV-HUC-1 were purchased from American Type Culture Collection (ATCC, USA). T24 and 5637 cells were cultured in RPMI-1640 (Gibco, Shanghai, China), RT4 cells were cultured in McCoy’s 5A(Gibco), UM-UC-3, J82, HT-1376, TCCSUP and HEK-293 T cells were cultured in DMEM (Gibco), whereas SV-HUC-1 cells were maintained in F-12 K media (Gibco), supplemented with 10% FBS (Gibco, USA) and 1% penicillin/streptomycin (Gibco). Cells were grown in a humidified atmosphere of 5% CO_2_ at 37 °C. For Actinomycin D assay, T24 and UM-UC-3 cells were exposed to 2 μg/mL Actinomycin D (Sigma, USA) to block transcription for 8, 16 and 24 h.

### Nuleic acid preparation and quantitative real-time polymerase chain reaction (qRT-PCR)

Genomic DNA was isolated with MiniBEST Universal Genomic DNA Extraction Kit Ver.5.0 (Takara, Japan), and total RNA was isolated by using RNAiso Plus (Takara, Janpan) according to the manufacturer’s instructions. For RNase R digestion, 2 μg of total RNA was incubated for 30 min at 37 °C with or without RNase R (3 U/μg) (Epicentre Technologies, USA), and the treated RNA was subsequently purified with the RNeasy MinElute Cleanup Kit (Qiagen, Germany). The nuclear and cytoplasmic fractions were extracted using a PARIS™ Kit (Life Technologies, USA) according to the manufacturer’s protocol. cDNA was synthesized using the PrimeScript RT Reagent Kit (Takara, Japan), and RT-PCR was performed on a Quantstudio™ DX system (Applied Biosystems, Singapore) using TB Green Premix Ex Taq II (Takara, Japan). GAPDH and small nuclear U6 were used as internal controls. The sequence information of primers was listed in Additional file 1: Table S1.

### Oligonuleotide transfection

Small interfering RNA (siRNA), miRNA mimics, miRNA inhibitors and negative control oligos were purchased from GenePharma (Shanghai, China). The sequences were listed in Additional file 2: Table S2. Cell transfection was conducted by using Lipofectamine RNAiMax (Life Technologies).

### circRNA plasmid construction and stable transfection

Human circACVR2A cDNA was synthesized and cloned into the plenti-ciR-GFP-T2A vector (IGE Biotech Co, China) to construct overexpression plasmids. The constructs were confirmed by sequencing. Afterwards, plasmids were transfected into HEK293T cells to package lentivirus to infect T24 and UM-UC-3 cells. Cells were selected for 3 days with 2 μg/mL puromycin, and surviving cells were used as stable transfectants.

### Cell proliferation, wound healing, migration and invasion assays

For cell viability detection, the MTS (Promega, USA) colorimetric assay was used according to the manufacturer’s protocol. The transfected cells were seeded in 96-well plates at a density of 2000 cells per well. Then absorbance was measured at a wavelength of 492 nm for 5 days using Spark 10 M (Tecan, Austria).

For colony formation assay, the transfected cells were seeded in 6-well plates at a density of 1000 cells per well. Approximately 10 days later, the clones were washed with 1x PBS, fixed with methanol and stained with 0.1% crystal violet. The clones were then imaged and quantified.

For wound healing assay, wounds were made using 200 μL pipette tips (time 0 h) in the middle of the six-well plates, and then the cells were cultured with serum-free medium immediately. After 12 h (for T24) or 24 h (for UM-UC-3), cell migration was photographed and the distance was measured and normalized to the 0 h control as the relative migration rate for comparison.

For transwell assay, chambers (8 μm pore size, Costar) with Matrigel (BD Science, USA) were used for cell invasion assays and chambers without Matrigel were used for cell migration assays. Approximately 5 × 10^4^ cells were suspended in 200 μL serum-free medium and added to the upper chambers. A total of 600 μL of medium containing 10% FBS was placed into the lower chambers as a chemoattractant. After incubation for 8 h (for T24) and 20 h (for UM-UC-3), cells in the upper chamber were softly removed with cotton swabs and cells on the lower surface were fixed with methanol and stained with 0.1% crystal violet for photographing and counting.

### Western blot analysis

Proteins were extracted using RIPA lysis buffer supplemented with 1% proteinase inhibitor and quantified by a BCA kit (Thermo, USA). Equal amounts of proteins (30 μg) were separated by 10% SDS–PAGE and transferred to PVDF membranes (Millipore, USA). After blocking for 1 h with 5% skim milk powder at room temperature, membranes were incubated with primary antibodies specific to EYA4(1:200, Santa Cruz, USA), ID2(1:500, Santa Cruz, USA) and GAPDH(1:5000, Abcam, UK) at 4 °C overnight. The membranes were then incubated with HRP conjugated goat anti-mouse secondary antibody (1:5000, Abcam, UK) and visualized using the Immobilon™ Western Chemiluminescent HRP Substrate (Millipore, USA).

### Biotin-coupled probe pull-down assay

The pull-down assay was performed as previously described [20, 22]. In brief, the biotinylated circACVR2A probe and oligo probe (GenePharma, China) were incubated with M-280 Streptavidin magnetic beads (Invitrogen, USA) at room temperature for 2 h to generate probe-coated beads. Then approximately 1 × 10^7^ BC cells were harvested, lysed, sonicated and incubated with probe-coated beads at 4 °C overnight. After washing, the RNA complexes bound to the beads were eluted and extracted with RNeasy Mini Kit (Qiagen) and analyzed by qRT-PCR assay.

### Biotin-coupled miRNA capture

Stably expressed circACVR2A BCcells were transfected with biotinylated miRNA mimics or nonsense control (GenePharma, China) using Lipofectamine RNAiMax (Life Technologies) and incubated for 48 h. M-280 streptavidin magnetic beads were washed with lysis buffer and blocked with yeast tRNA on a low speed rotator at 4 °C for 2 h. The cells were harvested, lysed, sonicated and incubated with the blocked beads at 4 °C overnight. The bound RNAs were purified using the RNeasy Mini Kit (Qiagen) and the abundance of circACVR2A in bound fractions was evaluated by qRT-PCR assay.

### Fluorescence in situ hybridization (FISH)

The Cy3-labeled circACVR2A probe and Cy5-labeled miRNA-626 probe were designed and synthesized by GenePharma (Shanghai, China). The sequences of the probes were listed in Additional file 3: Table S3. The signals of the probe were detected by the Fluorescent In Situ Hybridization Kit (GenePharma, China) according to the manufacturer’s protocols. All images were acquired on ZEISS LSM800 Confocal Microscope system (Carl Zeiss AG, Germany).

### Luciferase reporter assay

HEK293T cells (5 × 10^4^) were seeded into 24-well plates and co-transfected with the corresponding psiCHECK™-2 vector and microRNA mimics. After incubation for 48 h, the firefly and Renilla luciferase activities were detected with a dual-luciferase reporter assay system (Promega, USA) according to the manufacturer’s instructions. Relative luciferase activity was normalized to the firefly luciferase internal control. Independent experiments were performed in triplicate.

### Haematoxylin and eosin (HE) staining and immunohistochemistry (IHC) analysis

This experiment was conducted as described previously [23, 24]. Primary antibodies specific for EYA4 (Santa Cruz, USA) and ID2 (Santa Cruz, USA) were used at the appropriate dilution in the experiments. Tissue samples of 5-μm thick paraffin section were stained with HE and IHC. Images were captured using a Nikon Eclipse 80i system with NIS-Elements software (Nikon, Japan).

### Animal experiments

BALB/c nude mice (5–6 weeks old) were purchased from the Experimental Animal Center, Sun Yat-sen University. All of the animal care and experimental procedures were approved by the Institutional Animal Care and Use Committee of Sun Yat-sen University and were performed in accordance with established guidelines. For tumor growth study, ten mice were included in each group, and stably expressed circACVR2A or control UM-UC-3 cells (5 × 10^6^ cells per mouse) were injected subcutaneously into the left side of the dorsum. The size of the tumor was measured every week. Four weeks later, the mice were sacrificed and examined for tumor weight. For popliteal LN metastasis assay, lentivirus-transduced UM-UC-3 cells (5 × 10^5^ cells per mouse) that stably expressed firefly luciferase were inoculated into the footpads and ten mice were used in each group. After four weeks, the bioluminescence of the popliteal LNs was detected by an in vivo bioluminescence imaging system, and then the popliteal LNs were enucleated and measured. Tumor specimens and popliteal LNs were fixed and embedded in paraffin for HE staining and IHC analysis.

### Statistical analysis

Statistical analyses were conducted using SPSS19.0 (SPSS, Chicago, IL, USA) or GraphPad Prism7.0 (GraphPad Prism, Inc., La Jolla, CA, USA). Student’s t test (two-tailed) was applied to assess the statistical significance between two groups. Chi-square test was used to analyze the correlation between circACVR2A expression levels and clinicopathological features in BC. Overall survival (OS) curves were calculated with the Kaplan-Meier method and analyzed with the log-rank test. Data were presented as the mean ± standard error of the mean (SEM). *P* < 0.05 was considered statistically significant.

## Results

### Identification and characterization of circACVR2A in BC cells

**Fig. 1:**
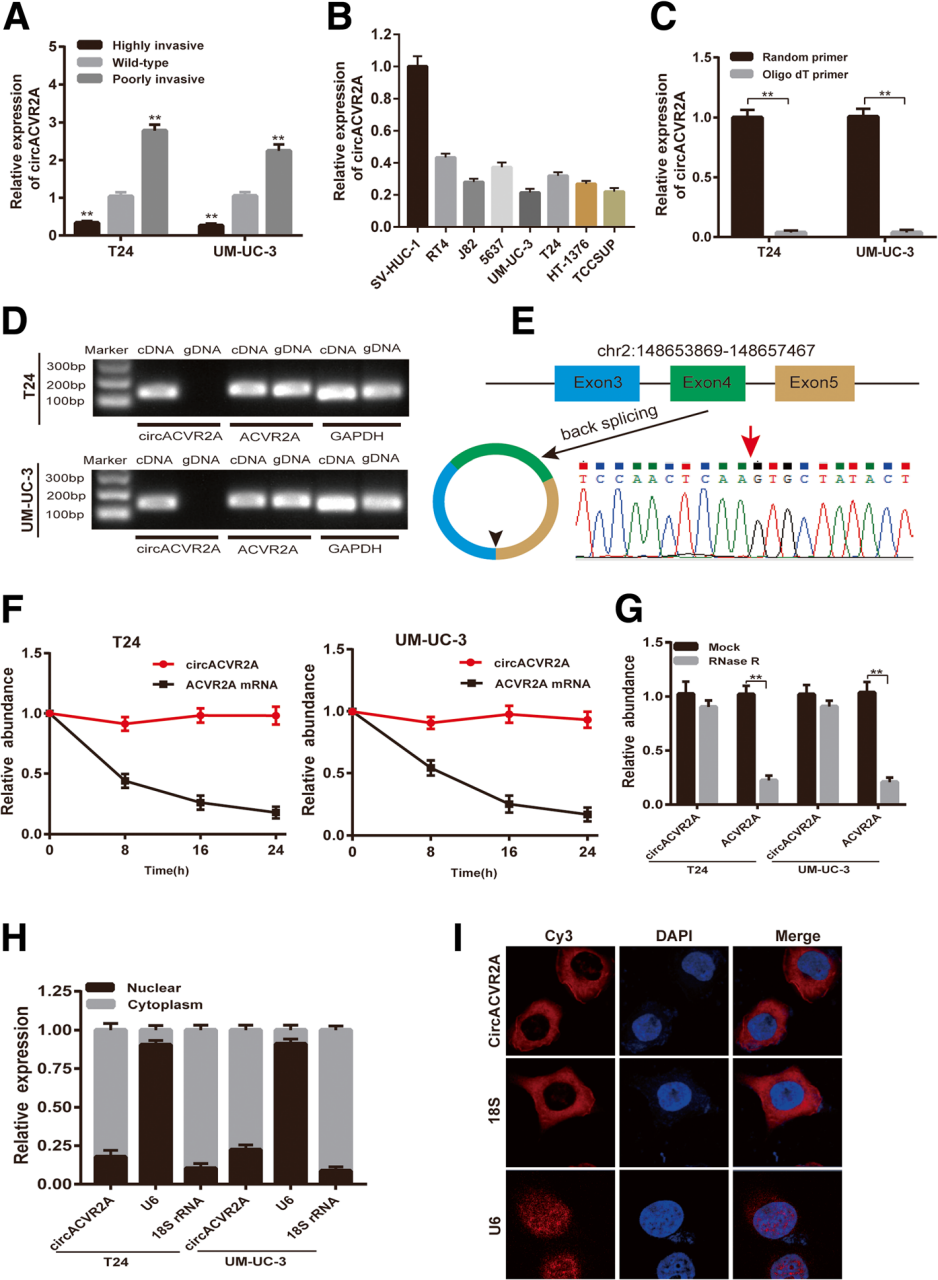
The validation and characteristics of circACVR2A in BC cells. **a** Relative expression of circACVR2A in our established poorly and highly invasive T24 and UM-UC-3 cell sublines. **b** Relative expression of circACVR2A in immortalized uroepithelium cell line SV-HUC-1, and BC cell lines RT4, J82, 5637, UM-UC-3, T24, HT-1376, TCCSUP. **c** qRT-PCR analysis of circACVR2A in the reverse transcription products using random primers or oligo dT primers. **d** The existence of circACVR2A was detected in T24 and UM-UC-3 cell lines by qRT-PCR with convergent or divergent primers and validated by Gel electrophoresis. **e** The expression of circACVR2A was validated by Sanger sequencing. Red arrow represents the back-splicing site of circACVR2A. CircAVCR2A derived from back-splicing of exons 3, 4 and 5 of ACVR2A gene. **f** qRT-PCR analysis of circACVR2A and ACVR2A mRNA after treatment with Actinomycin D at the indicated time points in T24 and UM-UC-3 cells. **g** qRT-PCR analysis of circACVR2A and ACVR2A mRNA after treatment with or without RNase R in T24 and UM-UC-3 cells. **h** qRT-PCR analysis of circACVR2A using nuclear and cytoplasmic fractions of T24 and UM-UC-3 cells. **i** FISH confirmed that circACVR2A was predominantly located in cytoplasm. Nuclei were stained with DAPI. U6, 18S and circACVR2A were labeled with Cy3

We firstly analyzed the published RNA-seq data of human BC tissues and paired normal bladder tissues [18] and found that circACVR2A was decreased in BC. We confirmed that circACVR2A expression was lower in highly invasive T24 and UM-UC-3 cell sublines than wild-type cells or higher in poorly invasive T24 and UM-UC-3 cell sublines than in wild-type cells using our previously established BC cell lines metastasis model [20, 21](Fig. 1a). Meanwhile, the expression of circACVR2A was significantly down-regulated in RT4, J82, 5637, UM-UC-3, T24, HT-1376 and TCCSUP BC cell lines, compared to the normal urothelial cell line SV-HUC-1(Fig. 1b). Since circRNAs don’t have 3′ polyadenylated tail, we detected the existence of circACVR2A in the reverse transcription products using random primers or oligo dT primers, and we verified that circACVR2A was almost undetectable when oligo-dT primers were used (Fig. 1c). Then, we designed divergent primers to amplify circACVR2A and convergent primers to amplify ACVR2A mRNA. CircACVR2A was only detectable in cDNA but not genomic DNA (gDNA) from T24 and UM-UC-3 cell lines by qRT-PCR with divergent primers, while ACVR2A could be amplified in both cDNA and gDNA using convergent primers (Fig. 1d). GAPDH was used as a control. Subsequently, Sanger sequencing of the qRT-PCR product of circACVR2A was performed to verify the head-to-tail splicing. The result was in accordance with circBase (http://circrna.org/), which indicated that circACVR2A was derived from exons 3, 4 and 5 of the ACVR2A gene (Fig. 1e).

Next, Actinomycin D assay demonstrated that the half-life of circACVR2A transcript exceeded 24 h, indicating that this isoform is more stable than the linear ACVR2A mRNA transcript in T24 and UM-UC-3 cells (Fig. 1f). Besides, RNase R digestion assay showed that the circular isoform was resistant to RNase R, whereas the linear isoform was obviously decreased after RNase R treatment (Fig. 1g). In addition, the subcellular localization of circACVR2A was detected with qRT-PCR analysis using nuclear and cytoplasmic fractions of T24 and UM-UC-3 cells and FISH assay. We found that circACVR2A was enriched in the cytoplasm fraction and mainly distributed in the cytoplasm (Fig. 1h-i). Taken together, these results indicated that circACVR2A was down-regulated in BC cell lines and was predominantly localized in the cytoplasm.

### CircACVR2A suppresses proliferation, migration and invasion of BC cells in vitro

**Fig. 2:**
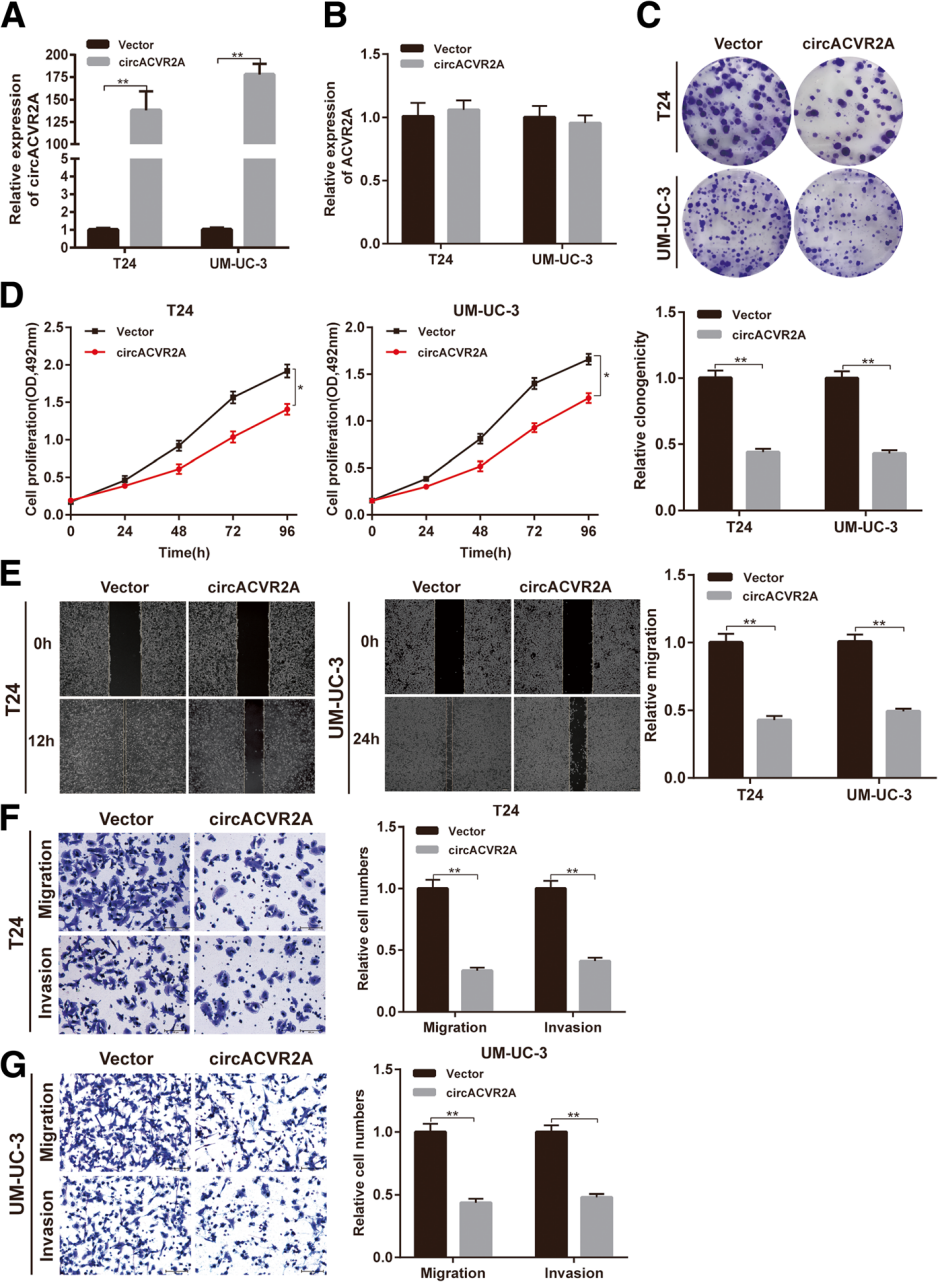
Overexpression of circACVR2A suppresses BC cells proliferation, migration and invasion in vitro. **a-b)** qRT-PCR analysis of circACVR2A and ACVR2A mRNA in T24 and UM-UC-3 cells after stable transfection of circACVR2A or vector. **c-d** Cell proliferation ability of T24 and UM-UC-3 cells transfected with circACVR2A or vector was evaluated by colony formation assay and MTS assay. **e** Cell migration capability of T24 and UM-UC-3 cells transfected with circACVR2A or vector was evaluated by wound healing assays. **f-g** The influence on cell migration and invasion abilities of T24 and UM-UC-3 cells transfected with circACVR2A or vector was assessed by transwell migration and matrigel invasion assays

To investigate the potential biological effect of circACVR2A on BC cells, we established circACVR2A stably overexpressing cell lines via transfecting with circACVR2A vector. We also used RNA interference to silence the expression of circACVR2A in T24 and UM-UC-3 cells. The overexpression and knock-down efficiencies of circACVR2A were detected by qRT-PCR analysis (Fig. 2a, Additional file 4: Figure S1A). The expression level of ACVR2A was not affected by circACVR2A changes (Fig. 2b, Additional file 4: Figure S1B). Colony formation assay and MTS assay showed that overexpression of circACVR2A reduced the proliferation ability of T24 and UM-UC-3 cells (Fig. 2c-d). Simultaneously, wound healing assay revealed that overexpression of circACVR2A suppressed cell migration in T24 and UM-UC-3 cells. Accordantly, overexpression of circACVR2A also significantly inhibited BC cells migration and invasion in transwell migration and Matrigel invasion assays, respectively (Fig. 2e-g). In contrast, silencing circACVR2A promoted the proliferation, migration and invasion of BC cells (Additional file 4: Figure S1C-F). Taken together, these results suggested that circACVR2A functioned as a tumor suppressor via inhibiting proliferation, migration and invasion of BC cells in vitro.

### CircACVR2A serves as a miRNA sponge for miR-626 in BC cells

**Fig. 3:**
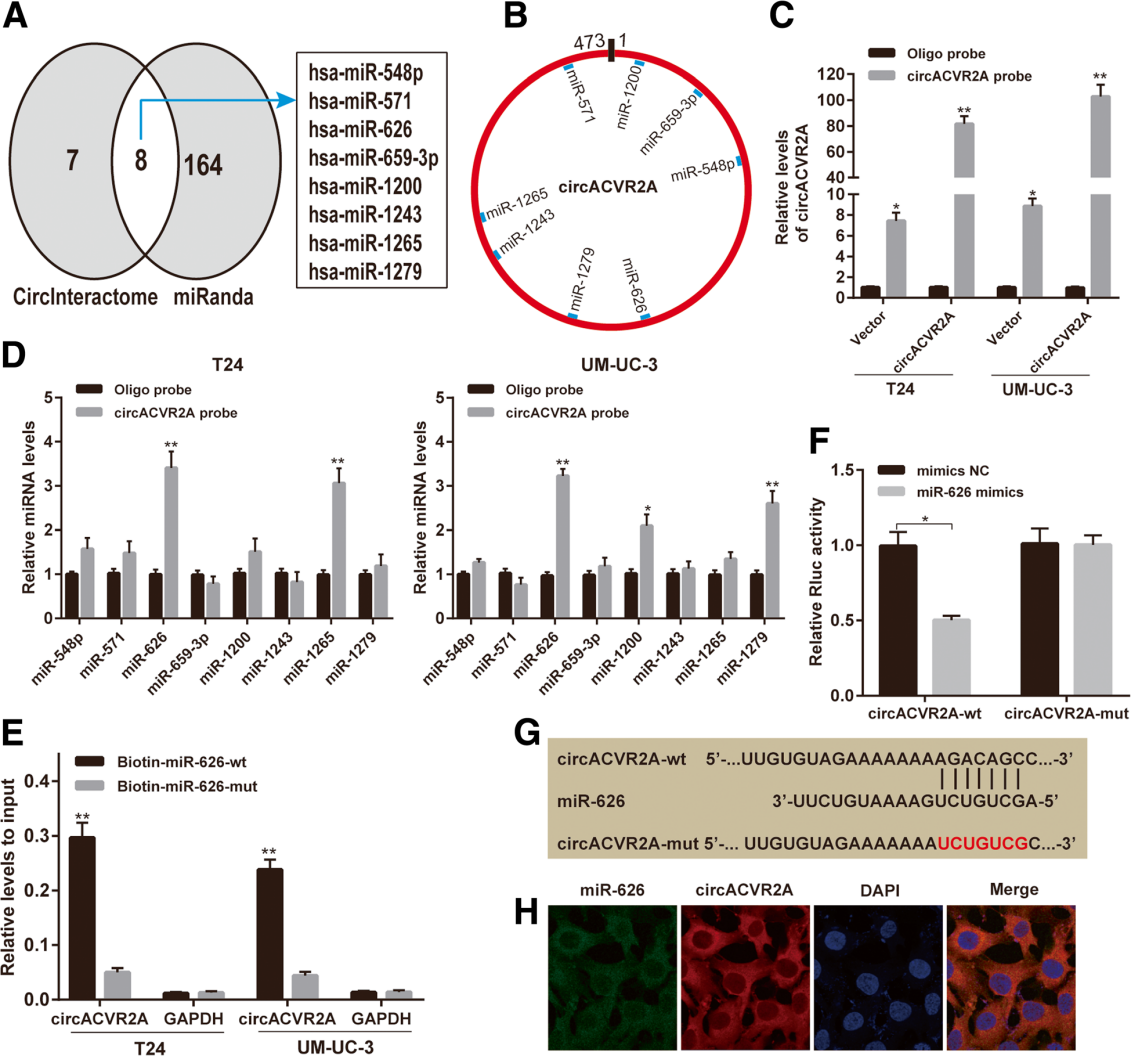
CircACVR2A serves as a miR-626 sponge in BC cells. **a** Eight potential target miRNAs of circACVR2A were predicted by CircInteractome and miRanda. (**b**)Schematic model showed the putative binding sites of eight miRNA candidates associated with circACVR2A. **c** Relative levels of circACVR2A in T24 and UM-UC-3 lysates after RNA pull down using circACVR2A probe or oligo probe. **d** Relative levels of eight miRNAs in T24 and UM-UC-3 lysates pulled down by circACVR2A probe or oligo probe. **e** Relative levels of circACVR2A in T24 and UM-UC-3 lysates captured by biotinylated wild-type miRNA-626 (Biotin-miR-626-wt) or mutant miR-626(Biotin-miR-626-mut). **f** Luciferase reporter assay in HEK293T cells co-transfected with miRNA mimics, psiCHECK-2-wild type circACVR2A (circACVR2A-wt) or psiCHECk-2-mutant type circACVR2A (circACVR2A-mut) plasmids. **g** Schematic of circACVR2A wild-type (wt) and mutant (mut) luciferase reporter vectors. **h** FISH showed the co-localization between circACVR2A and miR-626 in T24 cells. Nuclei were stained with DAPI. CircACVR2A was labeled with Cy3 and miR-626 was labeled with Cy5

Since circRNAs predominantly located in the cytoplasm were usually associated with miRNA sponging [25, 26], we further explored whether circACVR2A could bind to miRNAs. Eight potential target miRNAs (miR-548p, miR-571, miR-626, miR-659-3p, miR-1200, miR-1243, miR-1265, miR-1279) of circACVR2A were predicted by CircInteractome and miRanda, and these miRNAs were selected as candidate miRNAs for subsequent experiments (Fig. 3a). The positions of putative binding sites in circACVR2A were shown in Fig. 3b. The biotinylated circACVR2A probe and oligo probe were designed and were applied to perform RNA pull-down assay. The pull-down efficiency was verified in T24 and UM-UC-3 cells transfected with circACVR2A or vector (Fig. 3c). The miRNAs pulled down by biotinylated probes were purified and analyzed by qRT-PCR. Among the eight candidate miRNAs, only miR-626 could be abundantly pulled down by circACVR2A probe in both T24 and UM-UC-3 cells (Fig. 3d).

To further confirm the sponge effect between circACVR2A and miR-626, we conducted biotin-coupled miRNA capture assay, luciferase reporter assay and FISH. T24 and UM-UC-3 cells stably overexpressing circACVR2A were transfected with biotin-labeled miR-626 (biotin-miR-626-wt) or its mutant (biotin-miR-626-mut), and circACVR2A captured by miR-626 was evaluated by qRT-PCR. We found that the enrichment of circACVR2A in the captured fraction was much higher in biotin-miR-626-wt group than in biotin-miR-626-mut group (Fig. 3e), indicating that miR-626 could also bind to circACVR2A. Meanwhile, the sequence of wild-type circACVR2A or the sequence with mutant miR-626 binding sites was inserted into psiCHECK-2 vector to perform luciferase reporter assays. After co-transfected with miRNA mimics and psiCHECK-2 vector for 48 h in HEK293T cells, the Rluc activity was detected. Up-regulation of miRNA-626 could significantly decrease relative Rluc activity, suggesting that miR-626 could interact with circACVR2A (Fig. 3f-g). Moreover, FISH analysis in BC cells showed that circACVR2A and miR-626 were co-localized in the cytoplasm (Fig. 3h). Collectively, these results implied that circACVR2A might function as a competing endogenous RNA (ceRNA) through targeting miR-626.

### miR-626 is up-regulated and exerts an oncogenic role by targeting EYA4 in BC cells

**Fig. 4:**
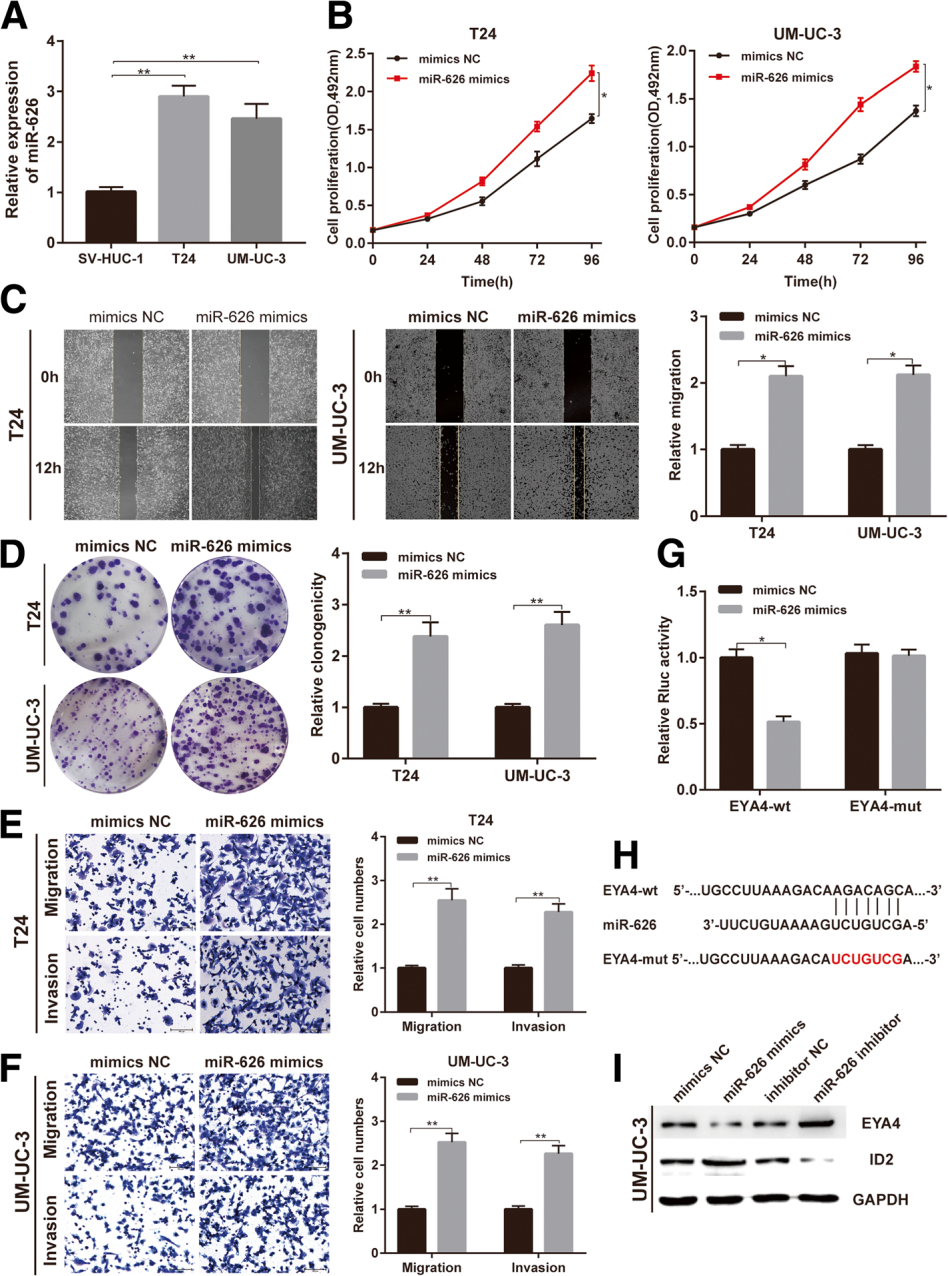
Up-regulated miR-626 promotes cell proliferation, migration and invasion through targeting EYA4 in vitro. **a** Relative expression of miRNA-626 in SV-HUC-1, T24 and UM-UC-3 cell lines. **b** Cell proliferation ability of T24 and UM-UC-3 cells transfected with mimics NC or miR-626 mimics was evaluated by MTS assay. **c** Cell migration capability of T24 and UM-UC-3 cells transfected with mimics NC or miR-626 mimics was evaluated by wound healing assays. **d** Cell proliferation ability of T24 and UM-UC-3 cells transfected with mimics NC or miR-626 mimics was evaluated by colony formation assay. **e-f** The influence on cell migration and invasion abilities of T24 and UM-UC-3 cells transfected with mimics NC or miR-626 mimics was assessed by transwell migration and matrigel invasion assays. **g** Luciferase reporter assay in HEK293T cells co-transfected with miRNA mimics, psiCHECK-2-wild type EYA4 (EYA4-wt) or psiCHECk-2-mutant type EYA4 (EYA4-mut) plasmids. **h** Schematic of EYA4 wild-type (wt) and mutant (mut) luciferase reporter vectors. **i** Western blot analysis indicated that miR-626 could down-regulate EYA4 and up-regulate ID2 expression in BC cells

Then, we evaluated the expression level and potential role of miR-626 in BC cells based on the association between circACVR2A and miR-626. The expression level of miR-626 was detected by qRT-PCR, and we found that miR-626 was obviously up-regulated in BC cell lines compared with SV-HUC-1 (Fig. 4a). Afterwards, we assessed the functional effect of miR-626 in T24 and UM-UC-3 cells by transfecting miRNA mimics and miRNA inhibitor. Cell proliferation, migration and invasion abilities were significantly promoted in BC cells transfected with miR-626 mimics as compared to the mimics NC group (Fig. 4b-f). On the contrary, inhibiting miR-626 significantly suppressed proliferation, migration and invasion capabilities of BC cells by transfecting miR-626 inhibitor (Additional file 5: Figure S2A and D).

We applied miRDB [27] and TargetScan [28] to identify the possible target genes of miR-626 in BC cells, and found that EYA4 could be a candidate target gene of miR-626. To testify this finding, we conducted luciferase reporter assays using psiCHECK-2 vector containing wild-type or mutant version of EYA4 3′ untranslated region (3’UTR). The Rluc activity was obviously reduced in HEK293T cells co-transfected with miR-626 mimics and EYA4-wt 3’UTR vector, but the Rluc activity was not distinctly changed in HEK293T cells co-transfected with miR-626 mimics and the mutant vector (Fig. 4g-h). Accordingly, Western blot analysis indicated that the expression of EYA4 protein was significantly decreased after transfection of miR-626 mimics, whereas EYA4 was up-regulated after transfection of miR-626 inhibitor in T24 and UM-UC-3 cells (Fig. 4i, Additional file 5: Figure S2E). Meanwhile, the expression level of ID2, a negative regulated downstream gene of EYA4 [29], had the opposite change compared to EYA4 level (Fig. 4i, Additional file 5: Figure S2E).

**Fig. 5:**
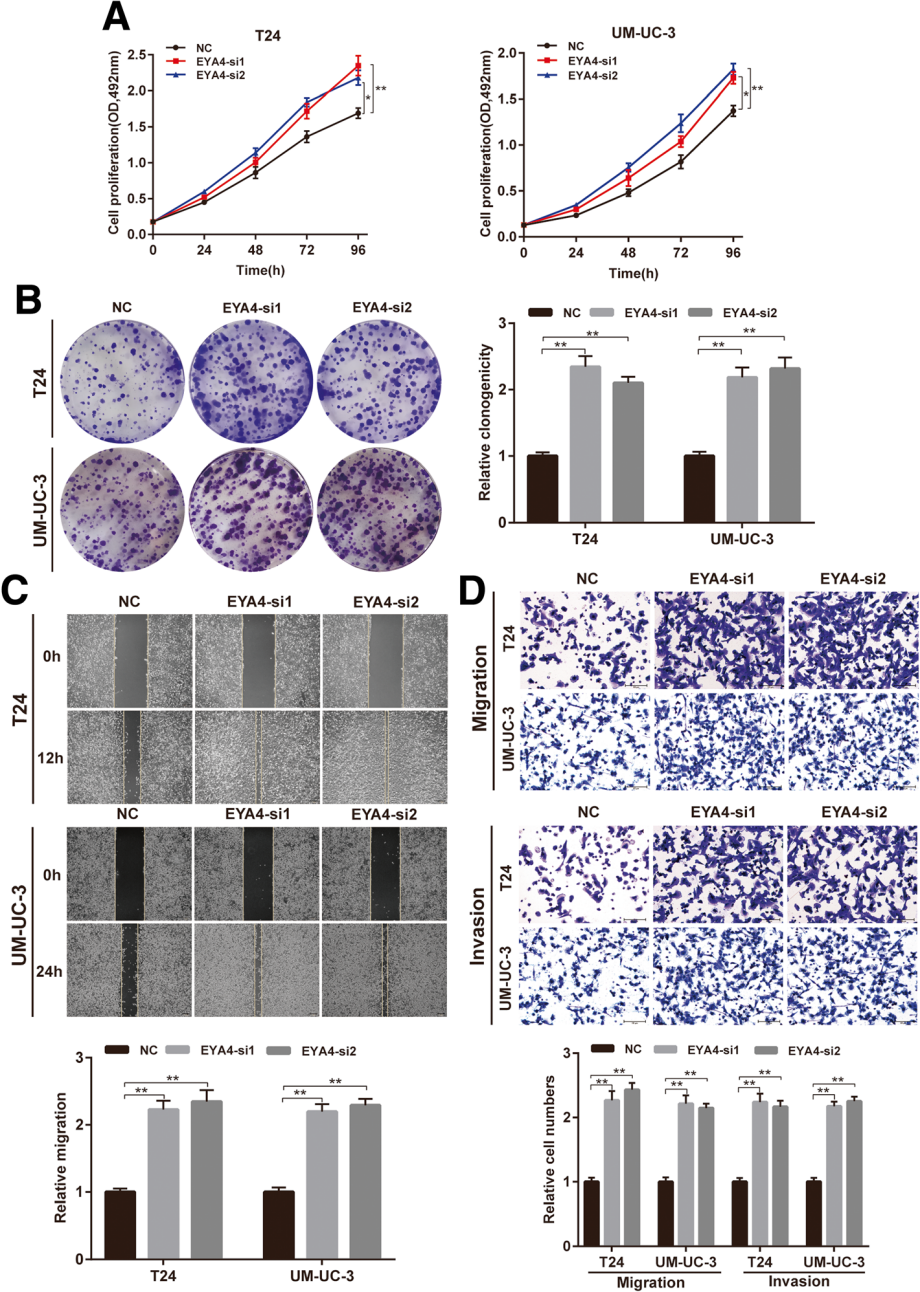
Down-regulation of EYA4 promotes proliferation, migration and invasion of BC cells in vitro. **a-b** Cell proliferation ability of T24 and UM-UC-3 cells transfected with EYA4 siRNAs was evaluated by MTS assay and colony formation assay. **c** Cell migration capability of T24 and UM-UC-3 cells transfected with EYA4 siRNAs was assessed by wound healing assays. **d** The influence on cell migration and invasion abilities of T24 and UM-UC-3 cells transfected with EYA4 siRNAs was evaluated by transwell migration and matrigel invasion assay, respectively

Previous studies have demonstrated that EYA4 acts as a tumor suppressor gene in non-small cell lung cancer [30], colorectal cancer [31], hepatocellular carcinoma [32], esophageal squamous cell carcinoma [33] and pancreatic ductal adenocarcinoma [29], but the biological role of EYA4 in BC remains unknown. Therefore, we designed two siRNAs targeting EYA4 to evaluate its influence on BC cells. Subsequent MTS assay and colony formation assay revealed that silencing of EYA4 promotes the growth of T24 and UM-UC-3 cells (Fig. 5a-b), and wound healing assay indicated that cell migration ability of BC cells transfected with EYA4 siRNAs was enhanced(Fig. 5c). In addition, down-regulation of EYA4 facilitated cell migration and invasion abilities assessed by transwell migration and matrigel invasion assays (Fig. 5d). These results showed that EYA4 exerted an anti-tumor effect on BC cells. Taken together, these data indicated that miR-626 promoted proliferation, migration and invasion of BC cells by targeting EYA4.

### CircACVR2A abrogates the oncogenic effect of miR-626 in BC cells

**Fig. 6:**
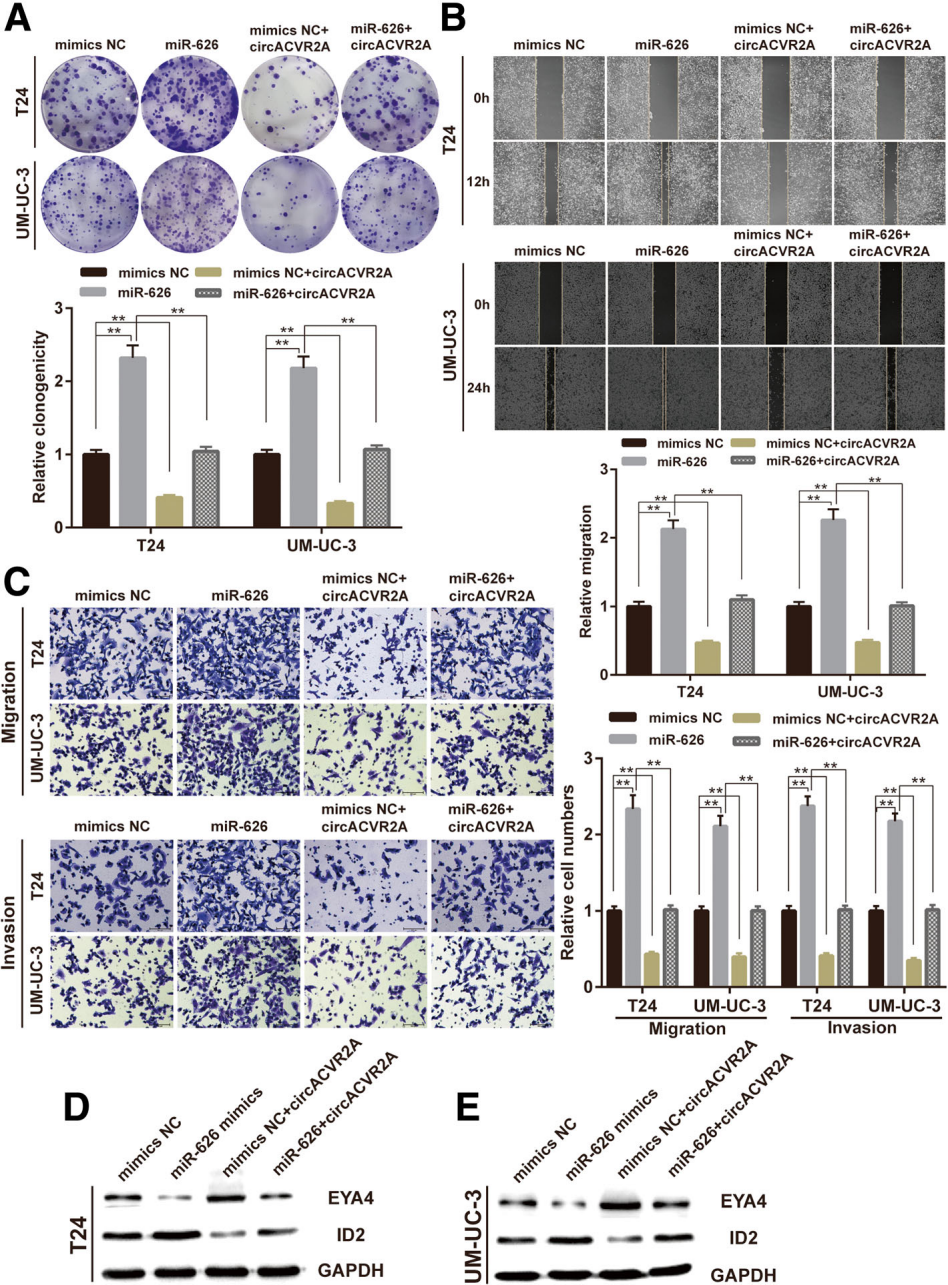
CircACVR2A reverses the oncogenic effect of miR-626 on BC cells in vitro. **a** Colony formation assay indicated that cell proliferation ability of T24 and UM-UC-3 cells transfected with miR-626 mimics was reversed when co-transfected with circACVR2A. **b** Wound healing assay indicated that cell migration capability of T24 and UM-UC-3 cells transfected with miR-626 mimics was reversed when co-transfected with circACVR2A. **c** Transwell migration and matrigel invasion assays demonstrated that cell migration and invasion abilities of T24 and UM-UC-3 cells transfected with miR-626 mimics were counteracted when co-transfected with circACVR2A.**d-e** Western blot analysis demonstrated that circACVR2A could counteract the influence of miR-626 mimics on EYA4 and ID2 expression in T24 and UM-UC-3 cells

Furthermore, rescue experiments were performed by co-transfecting circACVR2A and miR-626 mimics in BC cells to estimate whether circACVR2A exerts the tumor-suppressing effect on BC cells via sponging miR-626. We found that the colony formation ability of BC cells co-transfected with miR-626 mimics and circACVR2A was reduced compared with that of BC cells transfected only with miR-626 mimics, suggesting that overexpression of circACVR2A could partly abolish the enhancement of proliferation induced by miR-626(Fig. 6a). Similarly, up-regulation of circACVR2A could also partly attenuate the miR-626 mediated promotion of migration and invasion in T24 and UM-UC-3 cells (Fig. 6b-c). Meanwhile, Western blot assay indicated that the EYA4 protein level was partly increased in BC cells co-transfected with miR-626 mimics and circACVR2A compared to BC cells transfected only with miR-626 mimics, while the ID2 protein level showed the opposite response (Fig. 6d-e). Collectively, these results demonstrated that circACVR2A inhibited BC cells progression partly through impairing the oncogenic role of miR-626.

### CirACVR2A inhibits tumor growth and lymphatic metastasis in vivo

**Fig. 7:**
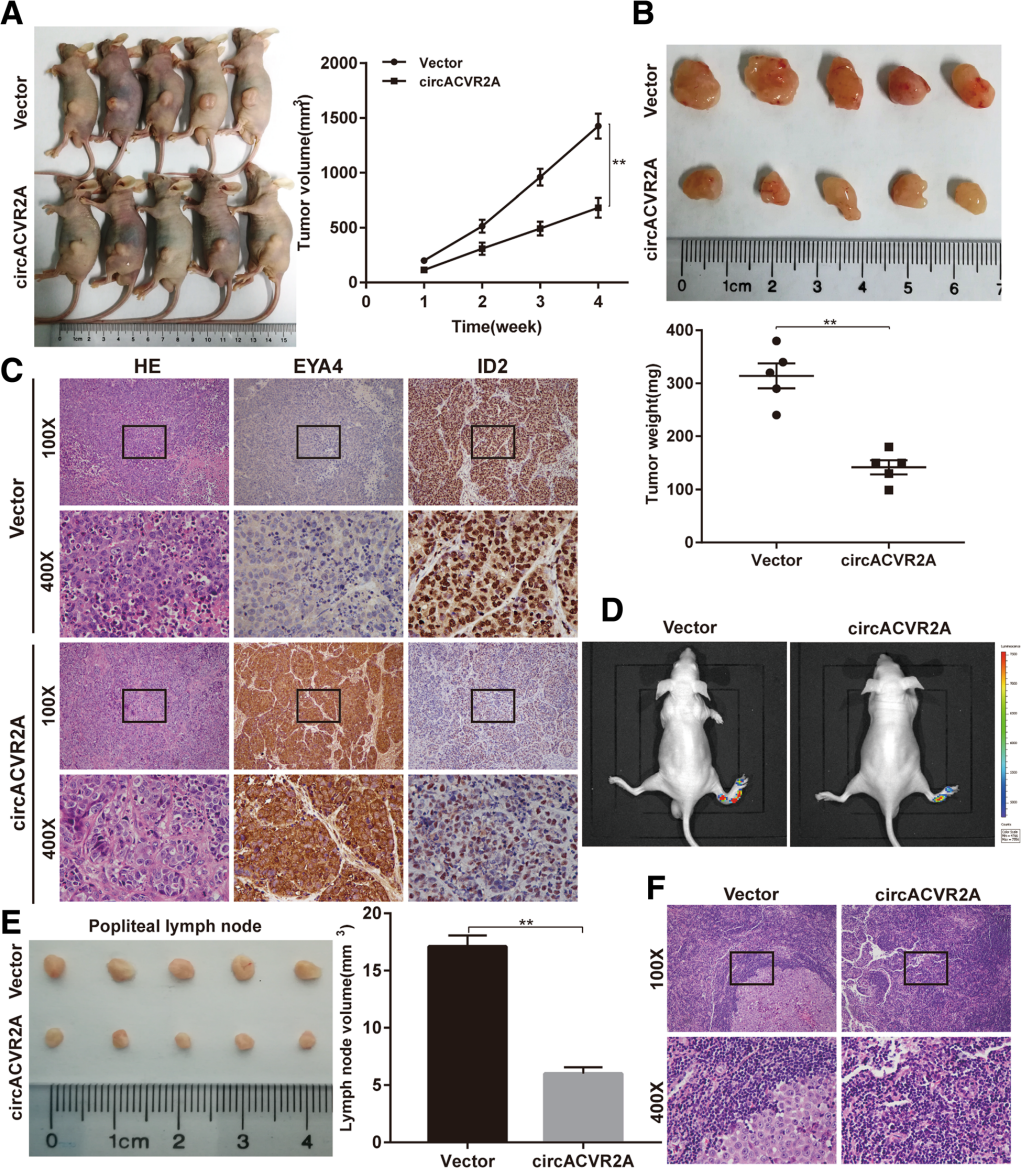
Overexpression of circACVR2A inhibits the growth and metastasis of BC cells in vivo. **a-b** UM-UC-3 cells stably transfected with circACVR2A or vector were injected subcutaneously into the left dorsum of BALB/c nude mice. Tumor volume and weight were dramatically decreased in circACVR2A overexpressing group. **c** HE staining and IHC analysis of EYA4 and ID2 expression in subcutaneous xenograft tumors. Overexpression of circACVR2A could up-regulate EYA4 and down-regulate ID2 expression. **d** Bioluminescence of the popliteal LNs was detected by an in vivo bioluminescence imaging system. **e** Popliteal LNs were enucleated and measured. The volume of popliteal LNs was significantly decreased in circACVR2A overexpressing group. **f** Representative images of HE staining analysis of the popliteal LNs in each group

To further investigate the effect of circACVR2A on tumor growth in vivo, UM-UC-3 cells stably transfected with circACVR2A or control vector were subcutaneously injected into BALB/c nude mice. The tumor volume and weight were significantly decreased in the circACVR2A overexpressing group compared with those of the vector group (Fig. 7a-b, Additional file 6: Figure S3A and B). IHC analysis was applied to detect the protein expression of EYA4 and ID2 in xenografted tumors of each group. The expression of EYA4 was obviously increased, while the ID2 expression level was remarkably reduced in tumor tissues of circACVR2A overexpressing group (Fig. 7c). Moreover, we also transfected firefly luciferase into UM-UC-3 cells previously constructed for xenograft, and those cells were injected into the footpads of nude mice. We found that the bioluminescence of popliteal LNs was weak or undetectable, and the volumes of popliteal LNs were obviously decreased in circACVR2A stably overexpressing group, suggesting that up-regulation of circACVR2A could suppress lymphatic metastasis of BC in vivo. (Fig. 7d-f, Additional file 6: Figure S3C).

### CircACVR2A is down-regulated in BC and associated with prognosis of BC patients

**Fig. 8:**
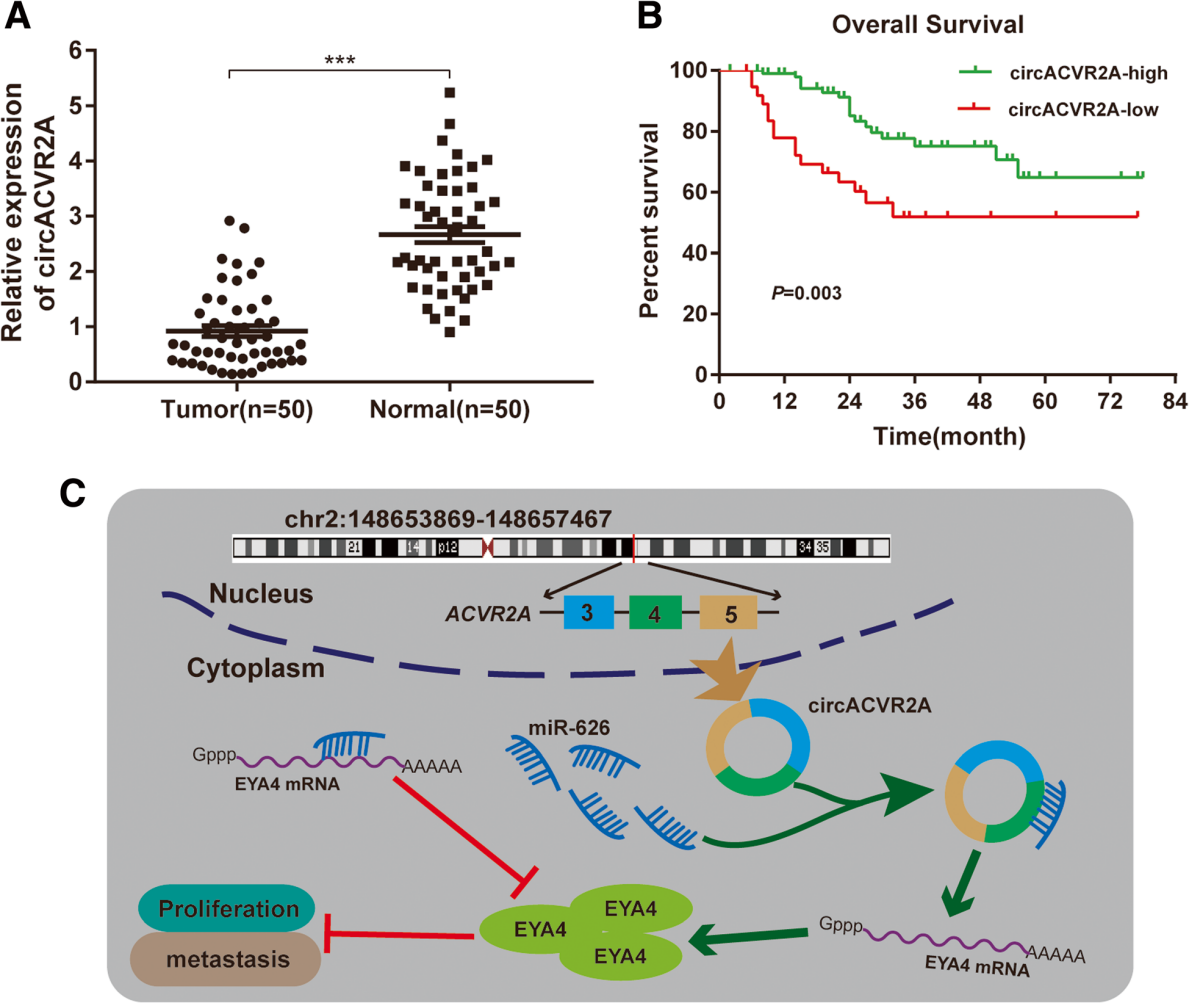
CircACVR2A is associated with prognosis of BC patients and exerts anti-tumor effects through miR-626/EYA4 axis. **a** The expression of circACVR2A in 50 pairs of BC tissues and adjacent normal tissues was detected by qRT-PCR analysis. **b** Kaplan-Meier survival curve demonstrated that low circACVR2A expression was correlated with low overall survival of BC patients. **c** Schematic diagram shows that circACVR2A inhibits BC cells proliferation and metastasis through miR-626/EY4 axis

We firstly applied qRT-PCR to detect the expression of circACVR2A in 50 pairs of BC tissues and matched adjacent normal tissues. We found that circACVR2A was significantly down-regulated in BC tissues compared with adjacent normal tissues (Fig. 8a). Subsequently, the expression of circACVR2A was evaluated in 140 cases of BC patients with complete survival data and clinical characteristics, and the results indicated that patients with advanced pathological T stage, high grade, positive lymphatic metastasis had low levels of circACVR2A, whereas other clinicopathological features were not significantly correlated with the expression of circACVR2A (Table 1). Furthermore, Kaplan-Meier analysis demonstrated that patients with low expression of circACVR2A were remarkably associated with poor OS (Fig. 8b).

**Table 1:** Correlations between circACVR2A expression levels and clinicopathological characteristics in BC

Characteristics		circACVR2A expression		*p*
	cases	Low (%)	High (%)	value
Age(years)				0.557
< 65	72(51.4)	18(25)	54(75)	
≥ 65	68(48.6)	20(29.4)	48(70.5)	
Gender				0.399
Male	103(73.6)	26(25.2)	77(74.8)	
Female	37(26.4)	12(32.4)	25(67.6)	
Pathology stage				0.016*
pTa-pT1	60(42.9)	10(16.7)	50(83.3)	
pT2-pT4	80(57.1)	28(35)	52(65)	
Grade				0.038*
Low	23(16.4)	2(8.7%)	21(91.3)	
High	117(83.6)	36(30.8)	81(69.2)	
Tumor size(cm)				0.591
< 3	118(84.3)	31(26.3)	87(73.7)	
≥3	22(15.7)	7(31.8%)	15(68.2)	
Lymph nodes metastasis				0.036*
Negative	109(77.9)	25(22.9)	84(77.1)	
Positive	31(22.1)	13(41.9)	18(58.1)	
Total	140	38	102	

^*^P < 0.05 was considered to be statistically significant (chi-square test)

## Discussion

Currently, an increasing number of circRNAs have been identified by means of bioinformatics analysis and high-throughput sequencing. Since circRNAs possess the regulatory potency of gene expression [7], and have been proven to be potential promising biomarkers [34], a large number of circRNAs have been investigated in the development and progression of different cancers [10, 11, 15, 35, 36], including BC [16–18, 20]. However, the functions of circRNAs in BC remain largely unknown, and still need to be further explored. In this study, we identified a new circRNA, circACVR2A, originating from exons 3, 4 and 5 of its host gene ACVR2A, that was down-regulated in T24 and UM-UC-3 cells and further decreased in our established highly invasive cell sublines [20]. Overexpression of circACVR2A significantly suppressed proliferation, migration and invasion of BC cells, whereas siRNA-mediated silencing of circACVR2A had the opposite effects on BC cells.

Accumulating studies have implied that circRNAs mainly act as miRNA sponges to exert various biological roles [26, 34, 37], thereby regulating downstream target genes. As the most well-known circRNA, ciRS-7 contains multiple miR-7 binding sites and decreases the biological effect of miR-7 on its target genes via sponging miR-7 [10, 26, 38]. In addition, emerging evidence has shown that the cytoplasmic localization of circRNA is closely associated with miRNA sponging [25, 26]. In our study, nuclear and cytoplasmic fractions assays and FISH confirmed that circACVR2A was predominantly distributed in the cytoplasm. Then, we verified that circACVR2A could interact with miR-626 in BC cells by biotinylated RNA pull-down and dual-luciferase reporter assays. We subsequently assessed the functional effects of miR-626 by transfecting miR-626 mimics or inhibitor into BC cells, and found that miR-626 exerted an oncogenic role on BC. Furthermore, overexpression of circACVR2A antagonized miR-626-mediated enhancement of cell proliferation, migration and invasion in BC cells. These results suggested that circACVR2A could serve as a miRNA sponge for miR-626.

Previous study has demonstrated that miRNAs can post-transcriptionally reduce the levels of specific target protein coding gene expression by binding to the 3’UTR of target mRNAs and resulting in translation inhibition or mRNA degradation [39]. Recent evidence indicates that circRNAs regulate gene expression by directly binding to miRNAs to prevent them from interacting with target genes [11, 18–20, 36]. In our study, EYA4 was predicted as the candidate target gene of miR-626 by miRDB and Targetscan, and was further testified by dual-luciferase reporter assay. Although several studies have shown that EYA4 acts as a tumor suppressor gene in some tumors [29–32], its association with BC has not been described. Therefore, our present study provided additional information to understand the biological function of EYA4 in BC cells. Moreover, we detected the expression of ID2, which has been identified as a negative regulated downstream gene of EYA4 [29]. The changes in ID2 expression suggested that it may be an oncogene in BC, which was consistent with a previous report [40].

Recent studies have indicated that circRNAs play a crucial role in the progression and prognosis of human cancer [37, 41]. The involvement of circRNAs in BC has been investigated in several studies. For instance, circPRMT5 promotes metastasis of BC through sponging miR-30c to induce EMT, and up-regulated expression of circPRMT5 was positively correlated with advanced stage and worse survival in BC patients [19]. Circ-ITCH inhibits BC progression by sponging miR-17/miR-224, and BC patients with low circ-ITCH had shortened survival [42]. In our study, we demonstrated that low expression of circACVR2A was associated with advanced pathological T stage, high grade, lymphatic metastasis and poor survival.

## Conclusions

In summary, our present study demonstrated that circACVR2A was down-regulated in BC cell lines and tissues, and its low expression was associated with poor clinicopathological characteristics of BC patients. Mechanistically, circACVR2A could significantly inhibit proliferation and metastasis of BC through directly binding to miR-626 and subsequently reduce the suppressing capability of miR-626 on EYA4, as shown in Fig. 8c. Our study suggested that circACVR2A was a novel potential prognostic biomarker and therapeutic target in BC.

## Additional files

Additional file 1: **Table S1.** The primers used in this study. (DOCX 14 kb)

Additional file 2: **Table S2.** The oligonucleotides transfected in this study. (DOCX 13 kb)

Additional file 3: **Table S3.** The probes used in this study. (DOCX 13 kb)

Additional file 4: **Figure S1.** CircACVR2A silencing promotes proliferation, migration and invasion of BC cells in vitro. (A-B) qRT-PCR analysis of circACVR2A and ACVR2A mRNA in T24 and UM-UC-3 cells after transfected with circACVR2A siRNAs. (C-D) Cell proliferation ability of T24 and UM-UC-3 cells transfected with circACVR2A siRNAs was evaluated by colony formation assay and MTS assay. (E) Cell migration capability of T24 and UM-UC-3 cells transfected with circACVR2A siRNAs was assessed by wound healing assays. (F) The influence on cell migration and invasion abilities of T24 and UM-UC-3 cells transfected with circACVR2A siRNAs was evaluated by transwell migration and matrigel invasion assay, respectively. (TIF 6721 kb)

Additional file 5: **Figure S2.** miRNA-626 exerts oncogenic effects on BC cells by targeting EYA4 in vitro. (A-B) Cell proliferation ability of T24 and UM-UC-3 cells transfected with inhibitor NC or miR-626 inhibitor was decreased using MTS assay and colony formation assay. (C) Cell migration capability of T24 and UM-UC-3 cells transfected with inhibitor NC or miR-626 inhibitor was suppressed using wound healing assays. (D) Cell migration and invasion abilities of T24 and UM-UC-3 cells transfected with inhibitor NC or miR-626 inhibitor were reduced using transwell migration and matrigel invasion assays. (E) Western blot analysis indicated that miR-626 could down-regulate EYA4 and up-regulate ID2 expression in BC cells. (TIF 5359 kb)

Additional file 6: **Figure S3.** Overexpression of circACVR2A suppresses the growth and metastasis of BC cells in vivo. (A-B) Tumor volume and weight were obviously decreased in circACVR2A overexpressing group. (C) The volume of popliteal LNs was significantly reduced in circACVR2A overexpressing group. (TIF 4305 kb)

## Abbreviations

‘UTR: 3’ untranslated region
ACVR2A: Activin A receptor type 2A
BC: Bladder cancer; circRNAs: circular RNAs
EMT: Epithelial-mesenchymal transition
EYA4: EYA transcriptional coactivator and phosphatase 4
ID2: Inhibitor of DNA binding 2
LN: Lymph node
qRT-PCR: Quantitative real-time polymerase chain reaction
