# Supplementary material for: Circular RNA ACVR2A suppresses bladder cancer cells proliferation and metastasis through miR-626/EYA4 axis
Source: Mol Cancer. 2019 May 17;18:95. doi: 10.1186/s12943-019-1025-z (PMC6524247; doi:10.1186/s12943-019-1025-z)
Supplement: Supplementary file 1 — Table S1. The primers used in this study. (DOCX 14 kb) [file 12943_2019_1025_MOESM1_ESM.docx]

|  | **Sequence (5’-3’)** |
| --- | --- |
| **Primers** | |
| circACVR2A Forward | AAGATGGCCTACCCTCCTGT |
| CircACVR2A Reverse | CCATAACACGGTTCAACACC |
| ACVR2A Forward | AAGCAAGGGGAAGATTTGGT |
| ACVR2A Reverse | ATGCTTCATTCCAGGCAAAC |
| GAPDH Forward | CAAGGCTGAGAACGGGAAG |
| GAPDH Reverse | TGAAGACGCCAGTGGACTC |
| EYA4 Forward | CTGGGTCAAAGTCCAGAGGA |
| EYA4 Reverse | CATAAGACCCGGTGAGCAGT |
| ID2 Forward | CCCAGAACAAGAAGGTGAGC |
| ID2 Reverse | ATAGTGGGATGCGAGTCCAG |
| miR-626 Forward | CCGGCGAGCTGTCTGAAAA |
| miR-548p Forward | CCGTAGCAAAAACTGCAGT |
| miR-571 Forward | CCGGTGAGTTGGCCATCTG |
| miR-659-3p Forward | CGCTTGGTTCAGGGAGGG |
| miR-1200 Forward | CCGCTCCTGAGCCATTCTG |
| miR-1243 Forward | CCGGAACTGGATCAATTATA |
| miR-1265 Forward | CCGCAGGATGTGGTCAAGT |
| miR-1279 Forward | CCCGGCGCGTCATATTGCTT |
| miRNA Reverse | GTGCAGGGTCCGAGGT |
| U6 Forward | CGCTTCGGCAGCACATATAC |
| U6 Reverse | TTCACGAATTTGCGTGTCAT |

**Table S1.** **The primers used in this study are listed as follows.**
