# Supplementary material for: Circular RNA ACVR2A suppresses bladder cancer cells proliferation and metastasis through miR-626/EYA4 axis
Source: Mol Cancer. 2019 May 17;18:95. doi: 10.1186/s12943-019-1025-z (PMC6524247; doi:10.1186/s12943-019-1025-z)
Supplement: Supplementary file 2 — Table S2. The oligonucleotides transfected in this study. (DOCX 13 kb) [file 12943_2019_1025_MOESM2_ESM.docx]

|  | **Sequence (5’-3’)** |
| --- | --- |
| **Oligonucleotides** | |
| si-NC sense | UUCUCCGAACGUGUCACGUTT |
| si-NC antisense | ACGUGACACGUUCGGAGAATT |
| circACVR2A-si1 sense | UCAAGUGCUAUACUUGGUATT |
| circACVR2A-si1 antisense | UACCAAGUAUAGCACUUGATT |
| circACVR2A-si2 sense | CAACUCAAGUGCUAUACUUTT |
| circACVR2A-si2 antisense | AAGUAUAGCACUUGAGUUGTT |
| EYA4-si1 sense | GAGUGGACUUUCCCAAACUTT |
| EYA4-si1 antisense | AGUUUGGGAAAGUCCACUCTT |
| EYA4-si2 sense | GGAGCGUAUAUGACAUCGATT |
| EYA4-si2 antisense | UCGAUGUCAUAUACGCUCCTT |
| mimics NC sense | UUCUCCGAACGUGUCACGUTT |
| mimics NC antisense | ACGUGACACGUUCGGAGAATT |
| miR-626 mimics sense | AGCUGUCUGAAAAUGUCUU |
| miR-626 mimics antisense | GACAUUUUCAGACAGCUUU |
| inhibitor NC | CAGUACUUUUGUGUAGUACAA |
| miR-626 inhibitor | AAGACAUUUUCAGACAGCU |

**Table S2.** **The oligonucleotides transfected in this study are listed as follows.**
