# Supplementary material for: Circular RNA ACVR2A suppresses bladder cancer cells proliferation and metastasis through miR-626/EYA4 axis
Source: Mol Cancer. 2019 May 17;18:95. doi: 10.1186/s12943-019-1025-z (PMC6524247; doi:10.1186/s12943-019-1025-z)
Supplement: Supplementary file 3 — Table S3. The probes used in this study. (DOCX 13 kb) [file 12943_2019_1025_MOESM3_ESM.docx]

**Table S3. The probes used in this study are listed as follows.**

|  | **Sequence (5’-3’)** |
| --- | --- |
| **FISH Probes** | |
| Cy3-U6 | TTTGCGTGTCATCCTTGCG |
| Cy3-18S | CTTCCTTGGATGTGGTAGCCGTTTC |
| Cy3-circACVR2A | TCTGATCTACCAAGTATAGCACTTGAGTTGGAACAAGTACAG |
| Cy5-miR-626 | AAGACATTTTCAGACAGCT |
| **Biotin-coupled probes** | |
| Biotin-NC | CUGUACUUGUUCCAACUCAAGUGCUAUACUUGGUAGAUCAGA |
| Biotin-circACVR2A | UCUGAUCUACCAAGUAUAGCACUUGAGUUGGAACAAGUACAG |
| Biotin-miR-626-wt | AGCUGUCUGAAAAUGUCUU |
| Biotin-miR-626-mut | ACGACAGAGAAAAUGUCUU |
