# Supplementary figures and images for: Circular RNA ACVR2A suppresses bladder cancer cells proliferation and metastasis through miR-626/EYA4 axis
Source: Mol Cancer. 2019 May 17;18:95. doi: 10.1186/s12943-019-1025-z (PMC6524247; doi:10.1186/s12943-019-1025-z)

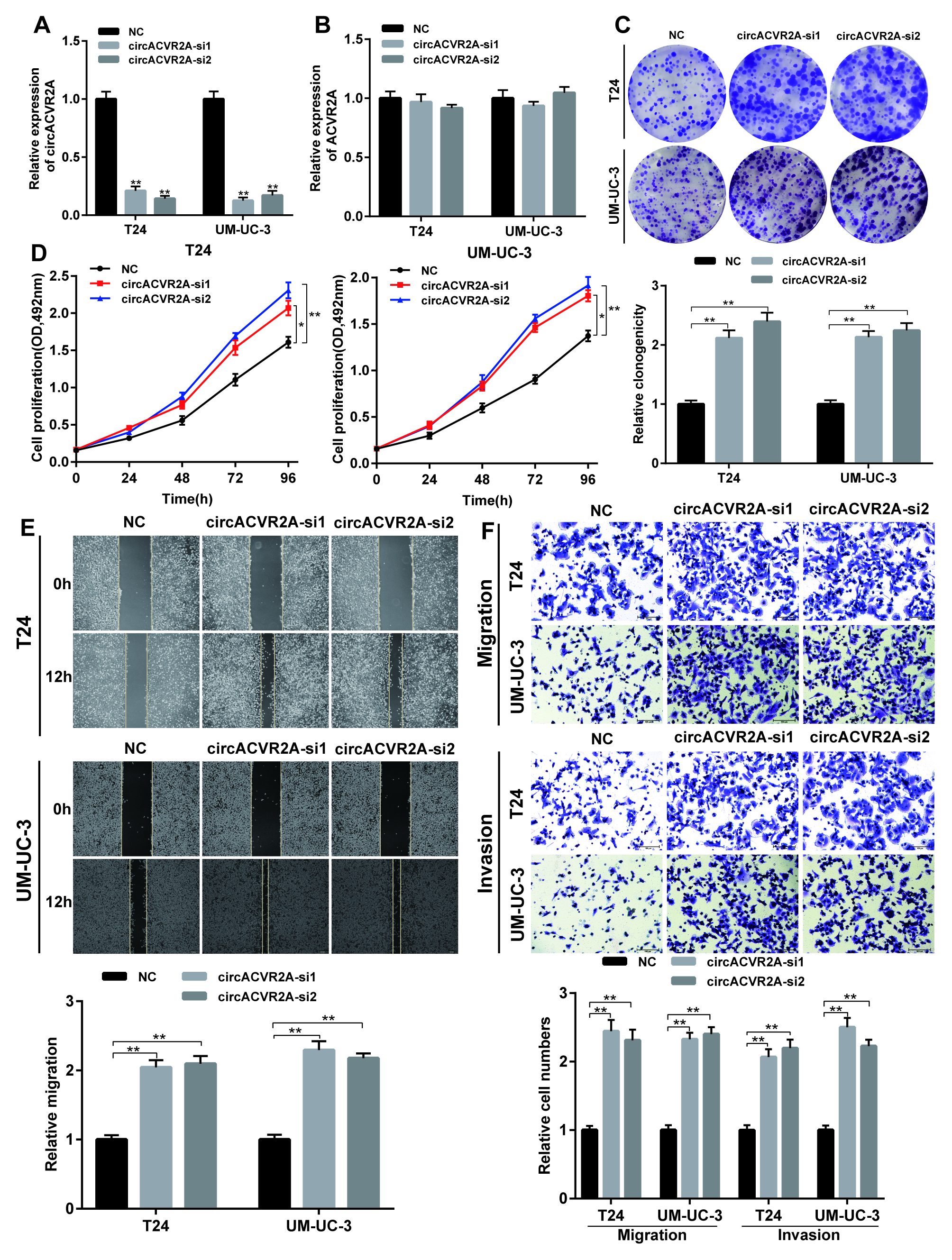

Supplement: Supplementary file 4 — Figure S1. CircACVR2A silencing promotes proliferation, migration and invasion of BC cells in vitro. (A-B) qRT-PCR analysis of circACVR2A and ACVR2A mRNA in T24 and UM-UC-3 cells after transfected with circACVR2A siRNAs. (C-D) Cell proliferation ability of T24 and UM-UC-3 cells transfected with circACVR2A siRNAs was evaluated by colony formation assay and MTS assay. (E) Cell migration capability of T24 and UM-UC-3 cells transfected with circACVR2A siRNAs was assessed by wound healing assays. (F) The influence on cell migration and invasion abilities of T24 and UM-UC-3 cells transfected with circACVR2A siRNAs was evaluated by transwell migration and matrigel invasion assay, respectively. (TIF 6721 kb) [file 12943_2019_1025_MOESM4_ESM.tif]

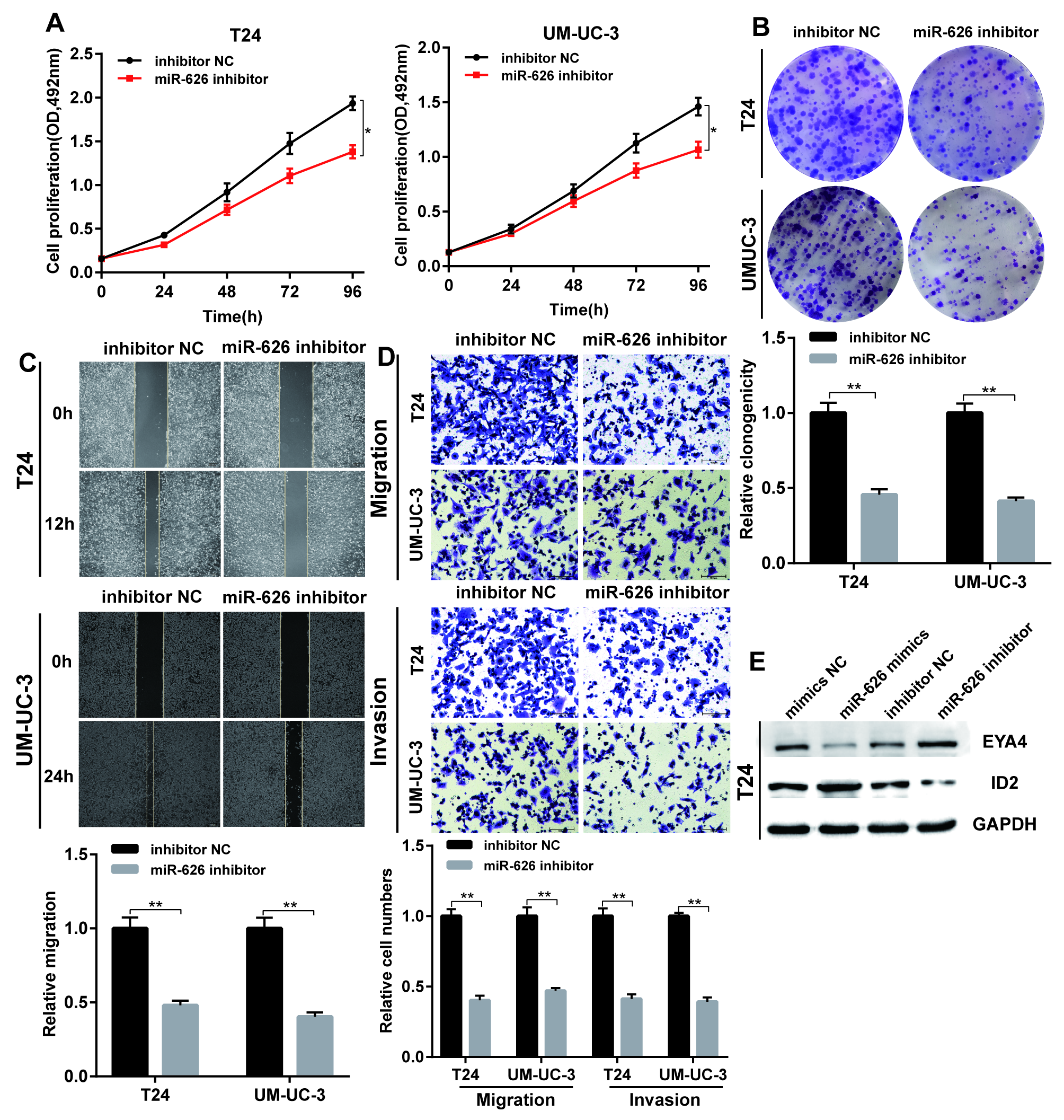

Supplement: Supplementary file 5 — Figure S2. miRNA-626 exerts oncogenic effects on BC cells by targeting EYA4 in vitro. (A-B) Cell proliferation ability of T24 and UM-UC-3 cells transfected with inhibitor NC or miR-626 inhibitor was decreased using MTS assay and colony formation assay. (C) Cell migration capability of T24 and UM-UC-3 cells transfected with inhibitor NC or miR-626 inhibitor was suppressed using wound healing assays. (D) Cell migration and invasion abilities of T24 and UM-UC-3 cells transfected with inhibitor NC or miR-626 inhibitor were reduced using transwell migration and matrigel invasion assays. (E) Western blot analysis indicated that miR-626 could down-regulate EYA4 and up-regulate ID2 expression in BC cells. (TIF 5359 kb) [file 12943_2019_1025_MOESM5_ESM.tif]

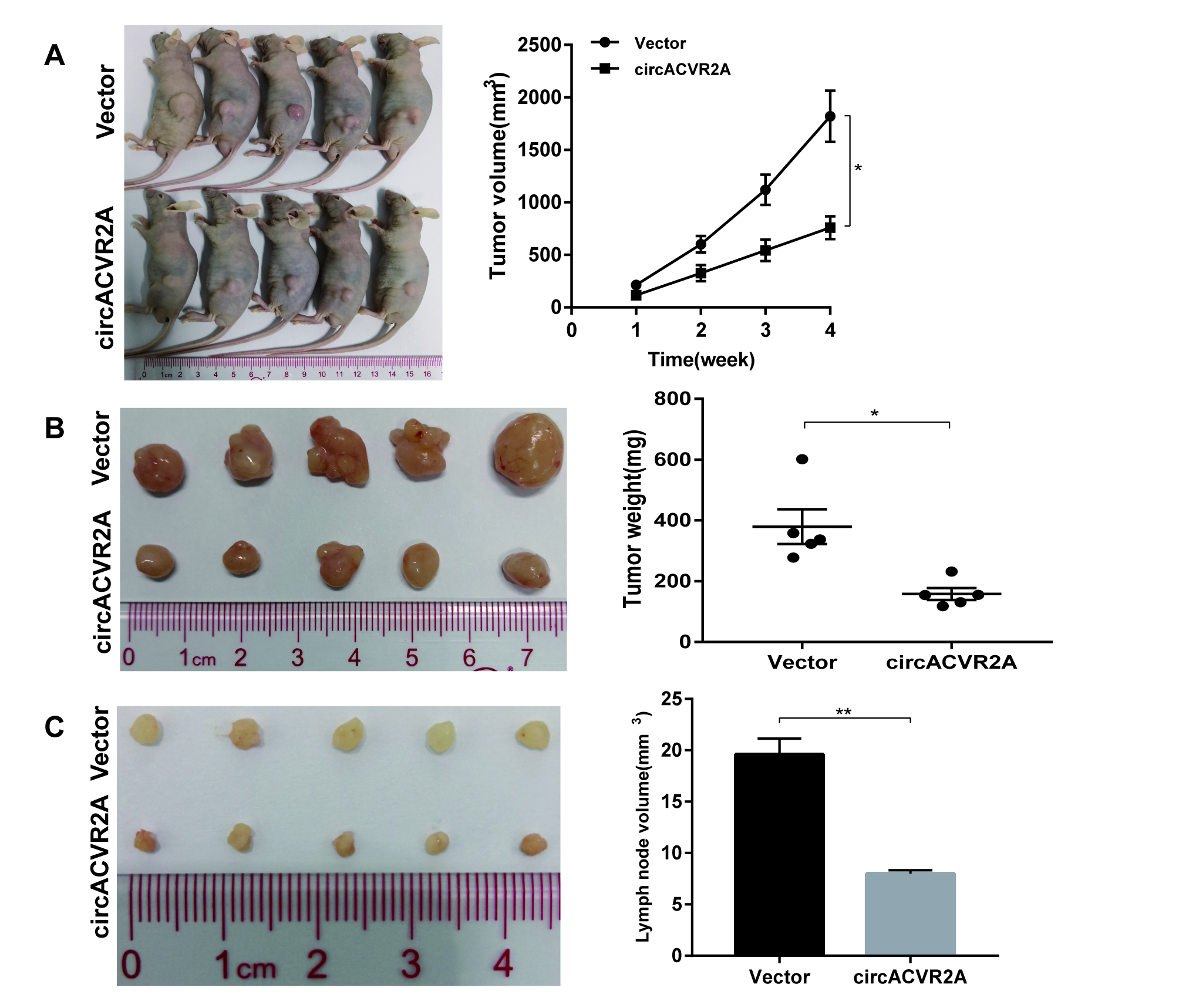

Supplement: Supplementary file 6 — Figure S3. Overexpression of circACVR2A suppresses the growth and metastasis of BC cells in vivo. (A-B) Tumor volume and weight were obviously decreased in circACVR2A overexpressing group. (C) The volume of popliteal LNs was significantly reduced in circACVR2A overexpressing group. (TIF 4305 kb) [file 12943_2019_1025_MOESM6_ESM.tif]
